# Differences in the clinical characteristics of chronic pulmonary aspergillosis according to spirometric impairment

**DOI:** 10.1371/journal.pone.0260274

**Published:** 2021-11-22

**Authors:** Myoung Kyu Lee, Sae Byol Kim, Beomsu Shin

**Affiliations:** 1 Department of Internal Medicine, Yonsei University Wonju College of Medicine, Wonju, Republic of Korea; 2 Division of Pulmonology, Department of Internal Medicine, Myongji Hospital, Jecheon, South Korea; 3 Division of Pulmonary and Critical Care Medicine, Department of Medicine, Samsung Changwon Hospital, Sungkyunkwan University School of Medicine, Changwon, Republic of Korea; Clinic for Infectious and tropical diseases, Clinical centre of Serbia, SERBIA

## Abstract

The clinical features by declining lung function remain uncharacterized in chronic pulmonary aspergillosis (CPA) patients. We investigated the clinical characteristics of CPA patients based on spirometric impairments (restrictive spirometric pattern [RSP] and obstructive spirometric pattern [OSP]) and their severity. We retrospectively analyzed medical records of CPA patients who underwent pulmonary function tests from March 2017 to February 2020. We used Global Lung Initiative 2012 equations with lower limit of normal. The clinical characteristics of patients with RSP were compared to those with OSP. Additionally, RSP patients’ characteristics were analyzed according to forced vital capacity (FVC) tertile, and OSP patients’ characteristics were analyzed according to forced expiratory volume in 1 second (FEV_1_) tertile. Among the 112 patients with CPA (52 [46%] with RSP and 60 [54%] with OSP), body mass index (BMI) was significantly lower in patients with RSP than in those with OSP (17.6 kg/m^2^ versus 20.3 kg/m^2^; *P* = 0.003), and non-tuberculous mycobacterial disease was more frequently observed in patients with RSP than in those with OSP (28.8% versus 11.7%; *P* = 0.004). Additionally, for patients with RSP, younger age and bilateral pulmonary lesions were more frequently observed in the first tertile group than in the other groups (P for trend: 0.025 and 0.001, respectively). For patients with OSP, low BMI, paracavitary infiltrates, and elevated WBC count were more frequently observed in the first tertile group than in the other groups (P for trend: < 0.001, 0.011, and 0.041, respectively). Differences in the clinical features of CPA patients were identified according to heterogeneous spirometric patterns and their severity. Further studies are needed to investigate the clinical significance of these findings.

## Introduction

Chronic pulmonary aspergillosis (CPA) is a progressively destructive disease caused by *Aspergillus* infection that results in inflammation and damage to the lung parenchyma and pleura [1]. CPA usually occurs in non-immunocompromised patients with pre-existing pulmonary diseases such as tuberculous destroyed lung, non-tuberculous mycobacterial disease (NTM), emphysema, bronchiectasis, and sarcoidosis [2, 3]. *Aspergillus* species grow in places where there are structural problems in the lungs [4]. While the progression of CPA is not fully understood, it is known that differences in the progression occur depending on the patient’s immune status, the condition of the underlying lung disease, and the severity of *Aspergillus* infection [5].

Abnormal spirometric results obtained from chronic lung disease may manifest in one of two forms, an obstructive spirometric pattern (OSP) or a restrictive spirometric pattern (RSP) [6]. Furthermore, pulmonary diseases with OSP are subdivided based on the forced expiratory volume in 1 second (FEV_1_), and those with RSP are subdivided based on the forced vital capacity (FVC) for the objective evaluation of clinical features and prognosis [7, 8]. Previous studies have demonstrated that spirometry was a useful method for measuring and monitoring chronic lung disease, which is divided into obstructive lung disease (e.g., chronic obstructive pulmonary disease [COPD] and bronchiectasis) and restrictive lung disease (e.g., interstitial lung disease [ILD] and sarcoidosis) [6].

However, unlike other chronic lung diseases, the clinical implication of spirometry results is not known in patients with CPA [9]. Therefore, we aimed to evaluate the clinical characteristics according to the spirometric patterns and severity of FVC or FEV_1_.

## Materials and methods

### Study population

Data were collected from consecutive patients with CPA who underwent PFT at the Wonju Severance Christian Hospital (an 866-bed, university-affiliated, tertiary referral hospital in Wonju, South Korea) between March 2017 and February 2020 and were retrospectively analyzed.

### Diagnosis of CPA

The diagnosis of CPA required a clinical decision by the combination of clinical, radiological, and microbiological parameters as follows: (1) compatible chronic respiratory symptoms including at least cough, sputum, breathlessness, or hemoptysis sustained for at least three months; (2) compatible chest radiological findings, including a cavity containing one or more aspergillomas or irregular intraluminal material with evidence of radiological progression (e.g., expansion of the cavity size, new cavities, or increasing paracavitary infiltrates); and (3) positive serum anti-Aspergillus antibodies (*Aspergillus fumigatus* IgG ELISA kit; IBL International, Hamburg, Germany) or positive *Aspergillus* species cultures from respiratory samples [1, 10]. Simple aspergilloma and subacute invasive aspergillosis were excluded from the present study [11]. All patients were observed for cavitary lesions on the chest CT in the present study. Finally, 183 patients with chronic cavitary pulmonary aspergillosis were recruited.

### Pulmonary function test

Spirometry was performed by trained technicians using a Vmax 22 apparatus (CareFusion, Yorba Linda, CA, USA) according to recommendations set by the American Thoracic Society/European Respiratory Society guidelines [12]. The absolute values for FVC and FEV_1_ were measured and the percentage of predicted values for FVC, FEV_1_, and the lower limit of normal (LLN; values below the fifth percentile in healthy, non-smoking subjects [z-score of −1.64]) were calculated using a reference equation obtained from the Global Lung Initiative (GLI) 2012 recommendation [13]. A normal spirometric pattern (NSP) was defined as a post-bronchodilator FEV_1_/FVC ≥ LLN and FVC ≥ LLN. RSP was defined as a post-bronchodilator FEV_1_/FVC ≥ LLN and FVC < LLN. OSP was defined as a post-bronchodilator FEV_1_/FVC < LLN. For the statistical analysis, the severity of RSP was classified according to the FVC tertile: tertile 1 was an FVC < 49% of the predicted value, tertile 2 was 49% ≤ FVC < 63% of predicted value, and tertile 3 was an FVC ≥ 63% of the predicted value. The severity of OSP was classified according to the FEV_1_ tertile: tertile 1 was an FEV_1_ < 38% of the predicted value, tertile 2 was 38% ≤ FEV_1_ < 54% of the predicted value, and tertile 3 was an FEV_1_ ≥ 54% of the predicted value.

### Data collection

Clinical data were collected from electrical health records. All information for patients included demographic data, comorbidities, respiratory symptoms, image findings, and laboratory parameters was collected retrospectively. “Breathlessness” represents a modified Medical Research Council dyspnea score ≥ 2 [14]. "Bilateral lung lesions" was defined as a case with compatible radiological findings of *Aspergillus* in both lungs.

### Ethics approval

This study was conducted in accordance with the amended Declaration of Helsinki. The Institutional Review Board for Human Research at Yonsei University Wonju Severance Christian Hospital (CR-320141) and the Institutional Review Board at the Samsung Changwon Hospital (SMC202010007) approved the study. As this study was a retrospective evaluation, written informed consent from each patient was waived. All data collected from each patient were de-identified prior to analysis.

### Statistical analysis

All data are expressed as median and interquartile range for continuous and ordinal variables, or as numbers and percentages for categorical variables. Continuous and categorical variables were analyzed by Mann–Whitney U test and Chi-square or Fisher’s exact test, respectively. To test for linear trends, subjects were grouped into tertiles of the observed FVC (% predicted) in RSP and FEV_1_ (% predicted) in OSP. The statistical significance level was set at a *P*-value of < 0.05. All statistical analyses were performed using SPSS version 26.0 (IBM Co., Armonk, NY, USA) statistical software.

## Results

### Patient characteristics

**Fig 1:**
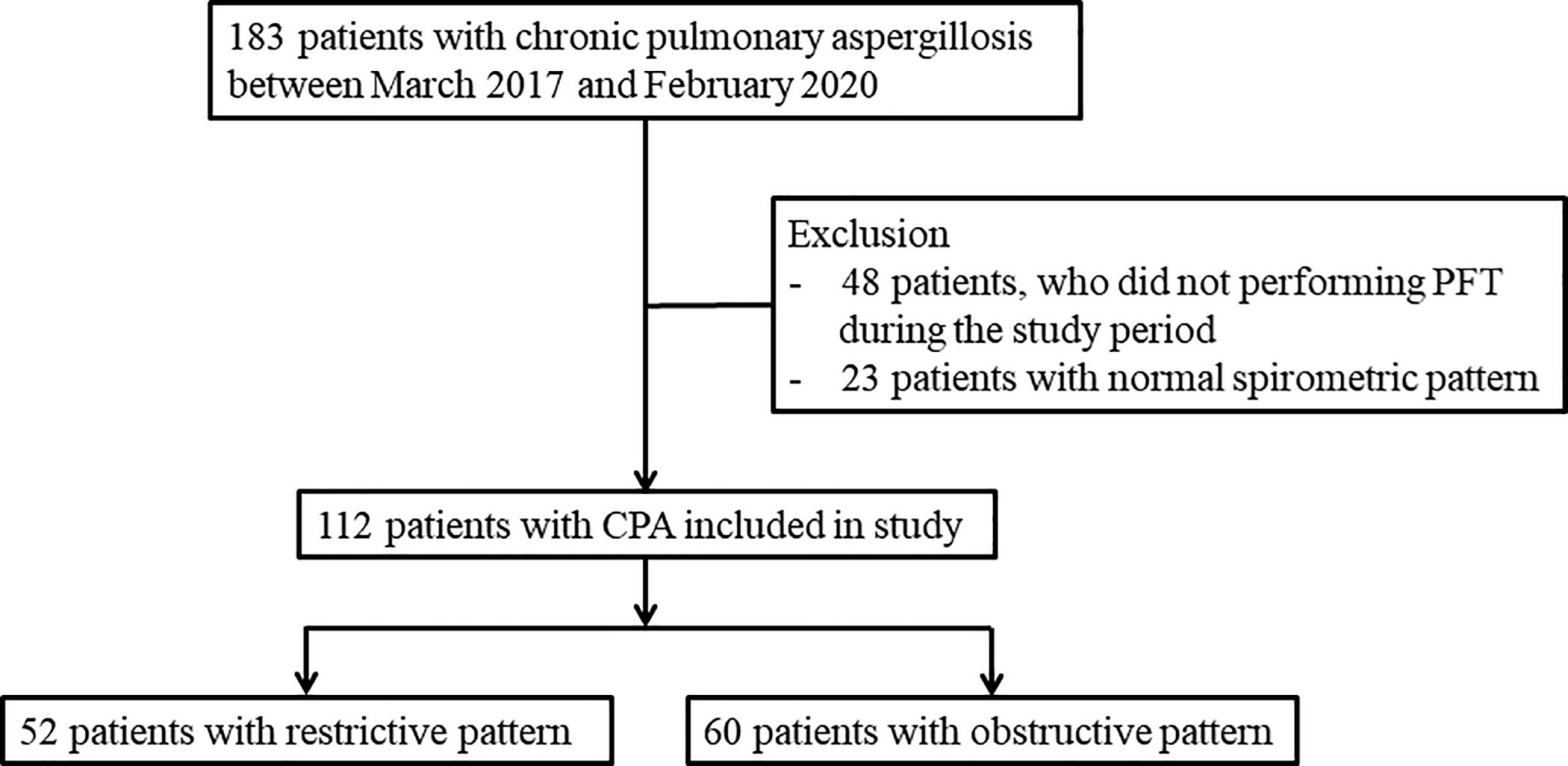
Flow chart of the study population in the study. PFT, pulmonary function test; CPA, chronic pulmonary aspergillosis.

A total of 183 patients were recruited. After excluding the patients who did not undergo pulmonary function test (PFT) (n = 48) or had NSP (n = 23), 112 patients were included in the study. The patients were further classified into RSP (n = 52, 46%) and OSP (n = 60, 54%) according to the spirometric patterns (Fig 1). The clinical characteristics of study participants are shown in Table 1. The median age of participants was 65 years and 87% of the participants were men. Seventy-six (68%) patients were current or ex-smokers. The main underlying diseases were tuberculous destroyed lung (n = 86, 77%), emphysema (n = 35, 31%), or NTM (n = 22, 20%). The most common respiratory symptoms were breathlessness (n = 66, 59%), cough (n = 56, 50%), sputum (n = 51, 46%), and hemoptysis (n = 34, 30%). All participants had at least one of the following imaging findings: paracavitary infiltration (n = 97, 87%), mycetoma (n = 44, 39%), or consolidation (n = 17, 15%). Bilateral pulmonary lesions were observed in 29 (26%) patients.

**Table 1:** Patient characteristics according to spirometric patterns^§^.

	Total	Restrictive spirometric pattern^§^	Obstructive spirometric pattern^§^	*P* value
	(N = 112)	(n = 52)	(n = 60)	
Age, years	65 (56–73)	64 (50–76)	65 (57–72)	0.966
Sex, male	87 (77.7)	42 (80.8)	45 (75.0)	0.503
Body mass index, kg/m^2^	19.5	17.6	20.3	**0.003**
	(16.8–21.8)	(16.3–20.6)	(17.4–22.5)	
Smoking history				
Ex or current smoker	76 (67.9)	32 (61.5)	44 (73.3)	0.225
Underlying lung disease*				
Previous history of pulmonary tuberculosis	86 (76.8)	41 (78.8)	45 (75.0)	0.660
Non-tuberculous mycobacterial disease	22 (19.6)	15 (28.8)	7 (11.7)	**0.031**
Emphysema	35 (31.3)	9 (17.3)	26 (43.3)	**0.004**
Bronchiectasis	16 (14.3)	8 (15.4)	8 (13.3)	0.792
Interstitial lung disease	3 (2.7)	3 (5.8)	0	0.097
Previous history of thoracic malignancy	3 (2.7)	2 (3.8)	1 (1.7)	0.596
Other comorbidities*				
Diabetes mellitus	14 (12.5)	5 (9.6)	9 (15.0)	0.568
Chronic hepatic insufficiency	10 (8.9)	3 (5.8)	7 (11.7)	0.334
Chronic renal insufficiency	1 (0.9)	0	1 (1.7)	> 0.999
Rheumatic disease	4 (3.6)	1 (1.9)	3 (5.0)	0.622
Previous history of extra-thoracic malignancy	12 (10.7)	5 (9.6)	7 (11.7)	0.769
Chronic pulmonary symptoms*				
Cough	56 (50.0)	28 (53.8)	28 (46.7)	0.570
Sputum	51 (45.5)	23 (44.2)	28 (46.7)	0.850
Breathlessness^†^	66 (58.9)	31 (59.6)	35 (58.3)	> 0.999
Hemoptysis	34 (30.4)	16 (30.8)	18 (30.0)	> 0.999
Chest computed tomographic findings*				
Cavitation	112 (100)	52 (100)	60 (100)	NA
Consolidation	17 (15.2)	6 (11.5)	11 (18.3)	0.430
Mycetoma	44 (39.3)	23 (44.2)	21 (35.0)	0.339
Paracavitary infiltrates	97 (86.6)	47 (90.4)	50 (83.3)	0.405
Bilateral pulmonary lesions	29 (25.9)	10 (19.2)	19 (31.7)	0.194
Laboratory findings				
White blood cells/μl	7,690	8,075	7,360	0.524
	(5,900–9,740)	(5,605–9,465)	(6,160–9,990)	
C-reactive protein, mg/dL	2.86	3.65	1.90	0.108
	(0.54–7.81)	(1.24–8.09)	(0.40–6.18)	
Albumin, g/dL	3.8	3.8	3.8	0.216
	(3.3–4.2)	(3.1–4.2)	(3.5–4.2)	

The data are presented as median (interquartile range) or number (%).

^§^ Spirometric pattern was defined as follows: (1) restrictive spirometry pattern was defined as a post-bronchodilator FEV_1_/FVC ≥ LLN and a FVC < LLN; (2) obstructive spirometry pattern was defined as a post-bronchodilator FEV_1_/FVC < LLN.

* Cases are duplicated.

^†^ “Breathlessness” represents a modified Medical Research Council dyspnea score ≥ 2.

FEV_1_, forced expiratory volume in 1 second; FVC, forced vital capacity; LLN, lower limit of normal.

Compared to the participants with OSP, the participants with RSP were more likely to have a lower body mass index (BMI) (17.6 kg/m^2^ versus 20.3 kg/m^2^; *P* = 0.003) and to have NTM (29% versus 12%; *P* = 0.031), but were less likely to have emphysema (17% versus 43%; *P* = 0.004). There were no significant differences in other clinical characteristics including pulmonary symptoms, chest CT findings, and laboratory findings between the two groups.

### Pulmonary function tests

PFT results are shown in Table 2. The median FVC and FEV_1_ were 2.12 L (62%) and 1.33 L (55%), respectively. The median FVC and FEV_1_ with RSP were 2.03 L (56%) and 1.78 L (71%), respectively, and the median FVC and FEV_1_ with OSP were 2.34 L (68%) and 1.05 L (46%), respectively.

**Table 2:** Pulmonary function tests.

	Total	Restrictive spirometric pattern^§^	Obstructive spirometric pattern^§^
	(N = 112)	(n = 52)	(n = 60)
FVC, L	2.12 (1.61–2.81)	2.03 (1.56–2.41)	2.34 (1.61–3.10)
FVC, % predicted	62 (48–76)	56 (43–68)	68 (53–87)
FEV_1_, L	1.33 (1.00–1.89)	1.78 (1.25–2.05)	1.05 (0.80–1.41)
FEV_1_, % predicted	55 (40–77)	71 (50–81)	46 (35–65)

The data are presented as median (interquartile range) or number (%).

^§^ Spirometric pattern was defined as follows: (1) restrictive spirometry pattern was defined as a post-bronchodilator FEV_1_/FVC ≥ LLN and a FVC < LLN; (2) obstructive spirometry pattern was defined as a post-bronchodilator FEV_1_/FVC < LLN.

FVC, forced vital capacity; FEV_1_, forced expiratory volume in 1 second; LLN, lower limit of normal.

### Clinical characteristics according to the spirometric patterns

Compared to the participants with RSP in FVC tertiles 2–3 (% predicted), those in tertile 1 were more likely to be younger (P for trend, 0.025) and have bilateral pulmonary lesions (P for trend, 0.001) (Table 3). However, there were no statistic differences in the trends for comorbidities, pulmonary symptoms, and laboratory findings among the three FVC tertile groups.

**Table 3:** RSP^§^ patients’ characteristics by tertile of FVC (% predicted).

FVC	Tertile 1	Tertile 2	Tertile 3	*P* for trend
(% predicted)	< 49	49 ≤–< 63	≥ 63	
	(n = 16)	(n = 19)	(n = 17)	
Age, years	56 (47–67)	68 (60–77)	68 (52–79)	**0.025**
Sex, male	12 (75.0)	16 (84.2)	14 (82.4)	0.825
Body mass index, kg/m^2^	17.3	17.3	19.3	0.051
	(15.2–21.2)	(16.4–19.3)	(16.9–22.1)	
Smoking history				
Ex or current smoker	9 (56.3)	11 (57.9)	12 (70.6)	0.723
Underlying lung disease*				
Previous history of pulmonary tuberculosis	14 (87.5)	13 (68.4)	14 (82.4)	0.401
Non-tuberculous mycobacterial disease	2 (12.5)	6 (31.6)	7 (41.2)	0.187
Emphysema	2 (12.5)	4 (21.1)	3 (17.6)	0.900
Bronchiectasis	3 (18.8)	3 (15.8)	2 (11.8)	0.895
Interstitial lung disease	0	2 (10.5)	1 (5.9)	0.766
Previous history of thoracic malignancy	1 (6.3)	1 (5.3)	0	0.756
Other comorbidities*				
Diabetes mellitus	1 (6.3)	1 (5.3)	3 (17.6)	0.502
Chronic hepatic insufficiency	2 (12.5)	0	1 (5.9)	0.192
Chronic renal insufficiency	0	0	0	NA
Rheumatic disease	1 (6.3)	0	0	0.308
Previous history of extra-thoracic malignancy	0	3 (15.8)	2 (11.8)	0.353
Chronic pulmonary symptoms*				
Cough	7 (43.8)	10 (52.6)	11 (64.7)	0.504
Sputum	6 (37.5)	8 (42.1)	9 (52.9)	0.733
Breathlessness^†^	9 (56.3)	12 (63.2)	10 (58.8)	0.939
Hemoptysis	6 (37.5)	5 (26.3)	5 (29.4)	0.810
Chest computed tomographic findings*				
Cavitation	16 (100)	19 (100)	17 (100)	NA
Consolidation	3 (18.8)	1 (5.3)	2 (11.8)	0.415
Mycetoma	8 (50.0)	7 (36.8)	8 (47.1)	0.778
Paracavitary infiltrates	16 (100)	17 (89.5)	14 (82.4)	0.302
Bilateral pulmonary lesions	8 (50.0)	1 (5.3)	1 (5.9)	**0.001**
Laboratory findings				
White blood cells/μl	7,275	8,610	7,135	0.454
	(5,643–9,213)	(5,773–10,310)	(5,093–9,143)	
C-reactive protein, mg/dL	7.94	2.67	3.49	0.055
	(3.52–12.23)	(0.59–9.23)	(0.78–4.62)	
Albumin, g/dL	3.5	4.0	3.9	0.794
	(3.1–4.1)	(3.2–4.4)	(3.0–4.1)	

The data are presented as median (interquartile range) or number (%).

^§^ Restrictive spirometry pattern was defined as a post-bronchodilator FEV_1_/FVC ≥ LLN and a FVC < LLN.

* Cases are duplicated.

^†^ “Breathlessness” represents a modified Medical Research Council dyspnea score ≥ 2.

RSP, restrictive spirometry pattern; FEV_1_, forced expiratory volume in 1 second; FVC, forced vital capacity; LLN, lower limit of normal.

Compared to participants with OSP in FEV_1_ tertiles 2–3 (% predicted), those in tertile 1 were more likely to have lower BMI (P for trend, < 0.001), paracavitary infiltrates (P for trend, 0.011), and higher average WBC counts (P for trend, 0.041) (Table 4). However, there were no statistic differences in trends for comorbidities and pulmonary symptoms among the three FEV_1_ tertile groups.

**Table 4:** OSP^§^ patients’ characteristics by tertile of FEV_1_ (% predicted).

FEV_1_	Tertile 1	Tertile 2	Tertile 3	*P* for trend
(% predicted)	< 38	38 ≤–< 54	≥ 54	
	(n = 18)	(n = 22)	(n = 20)	
Age, years	63 (58–71)	61 (55–68)	72 (59–78)	0.134
Sex, male	14 (77.8)	14 (63.6)	17 (85.0)	0.319
Body mass index, kg/m^2^	17.2	20.3	21.9	**< 0.001**
	(15.2–19.9)	(19.4–22.0)	(20.2–24.6)	
Smoking history				
Ex or current smoker	13 (72.2)	15 (68.2)	16 (80.0)	0.711
Underlying lung disease*				
Previous history of pulmonary tuberculosis	14 (77.8)	17 (77.3)	14 (70.0)	0.868
Non-tuberculous mycobacterial disease	4 (22.2)	1 (4.5)	2 (10.0)	0.233
Emphysema	9 (50.0)	8 (36.4)	9 (45.0)	0.721
Bronchiectasis	1 (5.6)	4 (18.2)	3 (15.0)	0.568
Interstitial lung disease	0	0	0	NA
Previous history of thoracic malignancy	0	0	1 (5.0)	0.633
Other comorbidities*				
Diabetes mellitus	3 (16.7)	5 (22.7)	1 (5.0)	0.287
Chronic hepatic insufficiency	2 (11.1)	3 (13.6)	2 (10.0)	> 0.999
Chronic renal insufficiency	0	1 (4.5)	0	> 0.999
Rheumatic disease	0	1 (4.5)	2 (10.0)	0.634
Previous history of extra-thoracic malignancy	1 (5.6)	3 (13.6)	3 (15.0)	0.687
Chronic pulmonary symptoms*				
Cough	10 (55.6)	10 (45.5)	8 (40.0)	0.653
Sputum	10 (55.6)	11 (50.0)	7 (35.0)	0.444
Breathlessness^†^	14 (77.8)	13 (59.1)	8 (40.0)	0.066
Hemoptysis	3 (16.7)	7 (31.8)	8 (40.0)	0.298
Chest computed tomographic findings*				
Cavitation	18 (100)	22 (100)	20 (100)	NA
Consolidation	4 (22.2)	3 (13.6)	4 (20.0)	0.770
Mycetoma	6 (33.3)	8 (36.4)	7 (35.0)	> 0.999
Paracavitary infiltrates	18 (100)	19 (86.4)	13 (65.0)	**0.011**
Bilateral pulmonary lesions	4 (22.2)	10 (45.5)	5 (25.0)	0.238
Laboratory findings				
White blood cells/μl	9,445	7,785	6,650	**0.041**
	(6,295–12,100)	(7,267–8,878)	(5,230–7,630)	
C-reactive protein, mg/dL	4.85	0.69	1.43	0.080
	(1.59–10.32)	(0.38–3.09)	(0.40–8.73)	
Albumin, g/dL	3.6	4.1	3.8	0.227
	(3.2–3.8)	(3.8–4.4)	(3.3–4.2)	

The data are presented as median (interquartile range) or number (%).

^§^ Obstructive spirometry pattern was defined as having a post-bronchodilator FEV_1_/FVC < LLN.

* Cases are duplicated.

^†^ “Breathlessness” represents a modified Medical Research Council dyspnea score ≥ 2.

OSP, obstructive spirometry pattern; FEV_1_, forced expiratory volume in 1 second; FVC, forced vital capacity; LLN, lower limit of normal.

## Discussion

CPA is a slowly progressive respiratory syndrome with obscure pathogenesis, complex methods of diagnosis, and limited therapeutic response [15, 16]. Thus, CPA can result in very diverse clinical outcomes. Until now, a pulmonologist can predict the condition and progress of the chronic lung diseases through PFT and imaging modalities [8, 17, 18]. However, the role of spirometry is not clear in patients with CPA [19, 20]. All participants in this study were classified based on pulmonary function results. Unlike other chronic diseases, patients with CPA have heterogeneous spirometric patterns. This was thought to be accompanied by various underlying diseases and the extent of lung damage caused by *Aspergillus* species, which are ubiquitous airborne molds [21–23].

In this study, the proportion of CPA patients with NTM was higher in patients with RSP than in those with OSP. This was due to the higher proportion of NTM patients with fibrocavitary form than those with nodular bronchiectatic form [24]. Conversely, the proportion of CPA patients with emphysema was higher in patients with OSP than in those with RSP [25]. Additionally, the BMI in the present study participants was in the normal range, but we found that CPA patients with RSP had a lower BMI than those with OSP. This was also considered to be due to the differences in the frequency of underlying diseases.

Among CPA patients with RSP (N = 52), the proportion of younger patients became higher as the FVC decreased. In the present study, the most common underlying lung disease in those patients is previous pulmonary tuberculosis (n = 41). The age was significantly lower in tuberculous destroyed lung patients with RSP in FVC tertile 1 compared to those in tertiles 2–3 (56 years versus 66 years; *P* = 0.025) (S1 Table). Additionally, although this was not statistically significant, the proportion of female participants with RSP in FVC in tertile 1 was higher than those in tertiles 2–3. The median age of women was 55 years while the median age of men was 68 years in the present study, with statistical significance (*P* < 0.001) (S2 Table). Although there are limitations in the interpretation due to the small number of patients included in our study, we were able to identify specific groups with decreased lung function among heterogeneous CPA patients. Further research is needed to investigate the differences in clinical features and prognosis for each group. The results also showed that the lower FVC, the higher frequency of bilateral lesions, which was an independent risk factor for CPA relapse [26]. This was thought to be because bilateral lesions themselves lead to FVC reduction.

Among CPA patients with OSP, on the other hands, the proportion of patients with lower BMI became higher as the FEV_1_ decreased. Previous research confirmed that only severe COPD was associated with underweight [27]. The study also confirmed that the lower FEV_1_, the higher frequency of paracavitary infiltrates with elevated WBC counts. These findings may indicate that paracavitary infiltrates by *Aspergillus* are associated with inflammatory aggravation, which could lead to the deterioration of FEV_1_.

Lastly, the spirometric results of CPA treatment response used in previous studies were based on FVC and FEV_1_ without categorization by spirometric patterns [14, 19, 28]. Our study showed that spirometric results categorized by spirometric patterns may be a viable alternative for monitoring the disease progression of CPA.

The current study had some limitations. First, this study was a retrospective analysis of patients from a single referral center and the sample size was relatively small, which might have led to selection bias. Second, not all patients underwent regular screening tests for CPA, mainly due to the low level of attention from pulmonologists and the complexity of the diagnostic method. Therefore, some CPA cases might have been missed. Third, PFT was not performed in all patients diagnosed with CPA during the study period. This is because it has not been recommended as a mandatory assessment in the previous guideline [1]. Spirometry might have been conducted more frequently in CPA patients with breathlessness, and these results could have affected the clinical characteristics. However, the present data would reflect real-world clinical practice.

## Conclusions

This study identified the difference in clinical features of the patients with CPA according to a variety of spirometric patterns, which possibly reflects the complexities of the patients with CPA. Further large-scale studies are required to evaluate the prognosis and mortality of CPA according to spirometric patterns.

## Supporting information

S1 Table Tuberculosis destroyed lung patients’ age by tertile of FVC (% predicted). The data are presented as median (interquartile range). FVC, forced vital capacity.
(DOCX)

S2 Table RSP^§^ patients’ age by tertile of FVC (% predicted) according to the sex. The data are presented as median (interquartile range). ^§^ Restrictive spirometry pattern was defined as a post-bronchodilator FEV_1_/FVC ≥ LLN and a FVC < LLN. RSP, restrictive spirometry pattern; FVC, forced vital capacity; FEV_1_, forced expiratory volume in 1 second; LLN, lower limit of normal.
(DOCX)

S1 Data (XLSX)

## List of abbreviations

BMI: body mass index
CPA: chronic pulmonary aspergillosis
COPD: chronic obstructive pulmonary disease
CT: computed tomography
FVC: forced vital capacity
FEV_1_: forced expiratory volume in 1 second
ILD: interstitial lung disease
LLN: lower limit of normal
NSP: normal spirometric pattern
OSP: obstructive spirometric pattern
PFT: pulmonary function test
NTM: non-tuberculous mycobacterial disease
RSP: restrictive spirometric pattern
