# Supplementary material for: Differences in the clinical characteristics of chronic pulmonary aspergillosis according to spirometric impairment
Source: PLoS One. 2021 Nov 22;16(11):e0260274. doi: 10.1371/journal.pone.0260274 (PMC8608325; doi:10.1371/journal.pone.0260274)
Supplement: S2 Table — The data are presented as median (interquartile range). § Restrictive spirometry pattern was defined as a post-bronchodilator FEV1/FVC ≥ LLN and a FVC < LLN. RSP, restrictive spirometry pattern; FVC, forced vital capacity; FEV1, forced expiratory volume in 1 second; LLN, lower limit of normal. (DOCX) [file pone.0260274.s002.docx]

**Supplementary Appendix**

**Differences in the clinical characteristics of chronic pulmonary aspergillosis according to spirometric impairment**

Myoung Kyu Lee^1^, Sae Byol Kim^2^, Beomsu Shin^3*^

^1^Department of Internal Medicine, Yonsei University Wonju College of Medicine, Wonju, Republic of Korea

^2^Division of Pulmonology, Department of Internal Medicine, Myongji Hospital, Jecheon, South Korea

^3^Division of Pulmonary and Critical Care Medicine, Department of Medicine, Samsung Changwon Hospital, Sungkyunkwan University School of Medicine, Changwon, Republic of Korea

^*^Corresponding author

E-mail: [bsshin83@gmail.com](mailto:bsshin83@gmail.com) (BS)

**S2 Table. RSP**^§^ **patients’ age by tertile of FVC (% predicted) according to the sex**

| Male | | | |
| --- | --- | --- | --- |
| FVC | Tertile 1 | Tertile 2 – 3 | *P* value |
| (% predicted) | < 49 | 49 ≤ |  |
|  | (n = 12) | (n = 30) |  |
| Age, years | 56 (47 – 61) | 72 (62 – 78) | **0.004** |
| Female | | | |
| FVC | Tertile 1 | Tertile 2 – 3 | *P* value |
| (% predicted) | < 49 | 49 ≤ |  |
|  | (n = 4) | (n = 6) |  |
| Age, years | 59 (44 – 71) | 50 (44 – 66) | 0.914 |

The data are presented as median (interquartile range).

^§^ Restrictive spirometry pattern was defined as a post-bronchodilator FEV_1_/FVC ≥ LLN and a FVC < LLN.

RSP, restrictive spirometry pattern; FVC, forced vital capacity; FEV_1_, forced expiratory volume in 1 second; LLN, lower limit of normal
